# Requests for physician-assisted suicide in German general practice: frequency, content, and motives– a qualitative analysis of GPs’ experiences

**DOI:** 10.1186/s12875-025-02830-0

**Published:** 2025-04-23

**Authors:** Luise Farr, Juliane Poeck, Claudia Bozzaro, Jutta Bleidorn

**Affiliations:** 1 Institute of General Practice and Family Medicine, Jena University Hospital, Friedrich Schiller University, Bachstr. 18, 07743 Jena, Germany; 2 Institute of Medical Ethics, History and Philosophy of Medicine, University of Münster, Münster, Germany

**Keywords:** Assisted suicide, Physician-assisted suicide, AS, PAS, Medical aid in dying, MAID, Primary care, GP practice, Germany, Experience, Motives, Frequency, Content, Interviews

## Abstract

**Background:**

Physician-assisted suicide (PAS) has been legalised in an increasing number of countries in Western Europe. In Germany, after a landmark decision by the Federal Constitutional Court in 2020, the ban on PAS was removed from the Model Medical Code of Conduct in 2021. Although the German Medical Association makes it clear that assisted suicide (AS) is not a genuine medical task, doctors have been approached about it. As long-standing, trusted companions of their patients, general practitioners (GPs) can be predestined as initial contacts for requests regarding PAS. Aim of this study is to assess the experiences of German GPs with requests for PAS.

**Methods:**

We conducted 19 guideline-based interviews with GPs currently or formerly practicing in Germany (study period: 03/22–12/22). The verbatim transcripts were analysed using Mayring’s qualitative content analysis.

**Results:**

In contrast to vague death wishes, requests for PAS were described as occasional. Nearly all respondents had experienced them several times. Most interviewees did not observe an increase in requests following the 2020 ruling by the Federal Constitutional Court. So far, the GPs’ role in PAS seems to be more of an advisory, informative, caring rather than an actively assisting one. According to the GPs’ reports most patients requesting PAS suffered from at least one form of cancer. Another significant group of patients was not severely ill but advanced in age. Regardless of age or illness, the interviewed GPs frequently perceived the loss of autonomy and independence as a primary motive for requesting PAS. Most of the requests involved either the plea for a lethal drug, information on the lethal dose of prescribed medication, or unspecified requests for assistance with suicide. Patients requesting PAS were predominantly described as educated, reflective, and financially well-off individuals.

**Conclusion:**

Individual insights into German GPs’ experiences with PAS suggest a high probability for GPs to encounter requests for PAS during their practice. Knowledge of vulnerable patient groups and prominent motives behind requests for PAS can be helpful in practice, enabling physicians to better understand and adequately respond to such requests.

**Supplementary Information:**

The online version contains supplementary material available at 10.1186/s12875-025-02830-0.

## Introduction

In recent years, there has been a trend toward the liberalisation of assisted suicide– including physician-assisted suicide– and euthanasia in Western Europe. These forms of aid in dying aim to enable individuals to die with self-determination. The critical distinction between these forms lies in the concept of control over the act. In euthanasia, another person administers a lethal substance to the individual wishing to die, typically upon his or her explicit request. This is usually carried out intravenously by a physician. In contrast, in assisted suicide (AS), the individual wishing to die carries out the life-ending act. Typically, this occurs through the oral or intravenous administration of a lethal substance, with the individual drinking the solution or activating the infusion himself or herself. The act of assistance often consists of making the lethal substance accessible and/or establishing venous access. If a physician performs the assistance, it is called physician-assisted suicide (PAS).

While euthanasia remains criminally prohibited in Germany and violations can result in imprisonment, AS has been a subject of public and political debate since the German Federal Constitutional Court *(Bundesverfassungsgericht)*, which is the highest court responsible for constitutional matters in the Federal Republic of Germany, declared the prohibition of the commercial facilitation of suicide unconstitutional in 2020 [1]. The court justified its decision by stating that the right to self-determined death is part of the right to personal freedom. This results in the unusual situation that, unlike in most other countries where AS is legally possible, in Germany neither a terminal illness nor unbearable suffering is required to access AS. The only criteria demanded in the ruling are that the wish for AS is persistent, well considered, and obtained self-responsibly, leaving open the question as how to ascertain any of them [1]. Although the Federal Constitutional Court suggested that the legislature implement a legal regulation [1], previous bills and proposed process regulations have failed [2]. Consequently, there are still no laws regulating AS in Germany. Legal guidance is therefore only possible based on the Federal Constitutional Court ruling [1], and criminal law judgments made since then.

According to the ruling, no special competencies are required to provide AS. Furthermore, the ruling makes clear that no individual nor professional group can be obliged to perform AS [1]. Nevertheless, physicians have been perceived as main potential actors in the ongoing discussion. Accordingly, in 2021, the ban of PAS was removed from the German Model Medical Code of Conduct *((Muster-)Berufsordnung für die in Deutschland tätigen Ärztinnen und Ärzte)* [3]. The Model Medical Code of Conduct itself is not legally binding but serves as a template for the Codes of Conduct of the State Medical Associations *(Landesärztekammern)*. To date, not all of them have adopted the removal of the prohibition on PAS. Thus, physicians in some federal states still face potential fines or professional sanctions for engaging in PAS. Furthermore, the German Medical Association *(Bundesärztekammer)* made it clear in a statement that AS is not a genuinely medical task and that the decision to participate in PAS is an individual matter of conscience [4].

Regardless of any legal or professional laws, German physicians have been confronted with requests for PAS in the past as well as in the present [5–8]. However, we know little about PAS and related requests in practice. An online reporting and learning system for the record of requests and experiences with AS and PAS in Germany has only recently been installed [9]. Knowledge about how often, by whom, to whom, and in what situations requests for AS and PAS are made can help improve the practical handling of such requests.

Regarding these questions, specialists in the fields of haematology/oncology [5, 6], palliative care [7], neurology, and psychiatry [10] have been in the centre of attention so far. Given that not only terminally ill individuals have access to PAS in Germany, it seems reasonable to also ask GPs about their experiences, attitudes, and needs. In the German healthcare system, GPs often are the first low-threshold point of contact for a wide range of health and psychosocial issues. GPs frequently accompany their patients over a long period of time. This creates the potential to get to know patients beyond individual diagnoses and to establish a special relationship of trust. As these characteristics predispose them to be confidants in sensitive matters, it is not surprising that even 20 years ago almost three quarters of German GPs received requests for PAS, almost all of them several times [8]. However, a recent study suggests that German GPs are not often involved in the practical implementation of PAS that are facilitated by right-to-die organisations [11]. For PAS occurring outside of right-to-die organisations, no data are available due to the lack of a specific ICD-10 code for (P)AS on death certificates and the absence of a well-established central registry until recently.

In other European countries where PAS is legal, the involvement of GPs in PAS varies according to legal and systemic circumstances. In contrast to the situation in Germany, a terminal illness or unbearable suffering is a mandatory prerequisite for being able to make use of PAS in other countries. Another difference between Germany and the Benelux countries is that euthanasia is permitted in the latter. In the Netherlands PAS and euthanasia have been established for many years and accounted for 5.4% of all deaths in 2023 [12]. GPs are the medical professionals most frequently involved in PAS or euthanasia in the Netherlands [12–14]. This includes all steps from request to implementation. Of approximately 9.000 reported cases of euthanasia and PAS in 2023, more than 7.000 were carried out and reported by GPs. Most of them took place at the patients’ home [12]. In Flanders, on the other hand, the majority of registered cases of euthanasia and PAS take place in clinics and GPs are generally not involved in implementation [14]. However, there is evidence that GPs in Flanders received requests for PAS and euthanasia even before they were legalised, and that some of these requests were granted [15].

In the German-speaking neighbouring countries Austria and Switzerland, GPs seem to be rather less involved. In Switzerland, right-to-die organisations have established themselves as the main points of contact [14]. Thus, it is not common, but still not unlikely for GPs to get requests from their patients to accompany them in a PAS [16, 17]. In 2014, a survey of 2.000 GPs showed that two-thirds did feel neither competent nor comfortable in handling such requests on their own [18] and tend to involve right-to-die organisations [16]. Even if an organisation is involved, most GPs are not ready to prescribe lethal drugs to their patients. Organisations have been keen to recruit more GPs as consultants [19].

In Austria, the Statute on the will to die *(Sterbeverfügungsgesetz)* has been in force since 2022. It regulates both eligibility criteria as well as the process leading towards PAS. This includes psychiatric and palliative consultations, as well as waiting times. Unless they are subspecialised in palliative care, GPs are not specifically mentioned in the law. A study from the time before the legalisation of PAS suggests that Austrian GPs receive requests for PAS [20]. First data from the Austrian **A**ssisted **S**uicide **C**riticial **I**ncident **R**eporting **S**ystem ASCIRS [21] do not allow any conclusions about the practical involvement of GPs [22].

Due to differences in the legal situations, healthcare systems, and societal acceptance and establishment of PAS, the transferability of international findings to Germany is not feasible. International studies can at best provide some inspiration in this question.

The HAPASS study (**Ha**usärztliche **P**erspektiven auf den **as**sistierten **S**uizid– GPs’ perspectives on (physician-)assisted suicide) explores the perspectives of German GPs on PAS. The aim of the present work is to provide insight into the experiences of German GPs with requests for PAS. The focus is on questions such as how often, for what reasons, and by whom GPs are being asked for PAS, what exactly they are being asked for, whether they have noticed changes since the Federal Constitutional Court’s ruling, and how they deal with implicit requests for PAS.

## Methods

### Study design

We conducted a qualitative survey with 19 GPs currently or formerly practicing in Germany. Interviews were conducted between March 2022 and December 2022. We aimed for theoretical saturation. Participants were recruited in a first outreach via email as a convenience sample from the Institute’s research and teaching practice network. Following an initial overview analysis, we expanded the study in a second outreach phase after consultation with CB. This was done with a call for participation in the GP journal *Der Allgemeinarzt* [23]. Participants should have worked as a GP in Germany for at least one year, either currently or in the past. With the exception of one participant, there was no personal relationship between participants and the interviewer. Out of 20 physicians who were either approached or responded to the call for participation, one interview could not be conducted due to time constraints.

The HAPASS study complies with the declaration of Helsinki. A positive ethics vote from the Institutional Research Ethics Board of the Jena University Hospital was obtained (Registration No.: 2022-2739-Bef.). All participants received written information about the study’s procedure, content, and objectives, and gave their written consent for the recording and further processing of the pseudonymised interview content. Participants received no incentives for participating in the study.

### Guideline-based interviews

We chose the method of guideline-based individual interviews to explore GPs’ perspectives on PAS. We developed the interview guideline based on the specific research questions, and agreed and piloted it with practicing GPs. During the individual interviews, an iterative process was followed. Based on their reported experiences, participants were asked more in-depth questions additionally to the guideline. The interview started with an open question about the interviewee’s points of contact with AS and PAS. In the further course, experienced frequencies of requests were enquired and individual experiences were explored in detail. Further questions concerned individual attitudes towards AS and PAS, the GP’s role in this topic, and perceived needs for a good handling of requests for PAS in general practice.

The research team had prior experience with qualitative interview studies. The lead author LF conducted the interviews. LF identifies as female and worked as a medical doctor and researcher at the time of the study. At the beginning of each interview, the interviewer verbally explained to the participants her academic interest in the study topic as a substantive connection between her training in general practice and her studies in applied ethics.

Interviews took place by phone or video conference and were audibly recorded. Participants were either at their workplace or at home during the interview. No one other than the interviewer and the participant was present during the interview. LF took field notes during and after each interview. No repeat interviews were carried out.

Records were transcript word for word, partially assisted by software (F4X), followed by a manual review by LF. All transcripts were pseudonymised. Transcripts were not returned to the participants. The German interview guideline and an English translation are provided in the Additional file.

### Data analysis

We performed Mayring’s qualitative content analysis [24] using MAXQDA 2022 software. In a first step, deductive main and subcategories were established based on the interview guideline. As new topics emerged during content analysis, we expanded the code system inductively (Table 1). Fourteen interviews were systematically coded and evaluated consensually by LF and CJ. Five additional interviews were coded by LF. In case of uncertainties, consultation, discussion, and consensus were sought with the research team (LF, CJ, JP). The presentation of methods and results follows the internationally recommended COREQ checklist [25].

**Table 1:** Category system

Main Categories	Subcategories	Type of Category Formation
1 Perceived frequency	1.1 Specific requests for PAS 1.2 Vague requests for assisted dying 1.3 Implicit requests for PAS	Deductive Deductive Inductive
2 Perceived Motives	2.1 Severe illness 2.2 Palliative situation 2.3 Pain 2.4 Other somatic symptoms 2.5 Psychological symptoms 2.6 Anxiety 2.7 Advanced age 2.8 Immobility 2.9 Loneliness 2.10 Loss of autonomy / Dependency 2.11 Weariness with life / Senselessness 2.12 Hopelessness 2.13 Survived own suicide attempt 2.14 Financial aspects 2.15 Immanent death	Deductive Deductive Deductive Deductive Deductive Deductive Deductive Inductive Deductive Deductive Deductive Deductive Inductive Inductive Inductive
3 Main diagnosis	3.1 Cancer 3.2 Neurological disease 3.3 Depression 3.4 Advanced age 3.5 Internal medical disease other than cancer 3.6 Chronic pain syndrome 3.7 Dementia 3.8 No medical diagnosis	Deductive Deductive Deductive Deductive Deductive Inductive Inductive Deductive
4 Content of requests	4.1 Request for lethal drugs 4.2 Request for information 4.3 Disclosure of information for right-to-die organisation 4.4 Referral abroad 4.5 Swallowing therapy 4.6 Unspecific	Deductive Deductive Inductive Deductive Inductive Deductive
5 Description of patients	5.1 Gender 5.2 Age 5.3 Character traits 5.4 Education / Occupation 5.5 Financial situation 5.6 Social situation 5.7 Biographical aspects	Deductive Deductive Inductive Inductive Inductive Deductive Inductive
6 Provided assistance with suicide	6.1 Intentional 6.2 Unintentional	Deductive Inductive
7 Perception of changes since ruling	7.1 Changes perceived 7.2 No changes perceived	Deductive Deductive

## Results

### Characteristics of participants

A total of 19 interviews were conducted, with an average recorded conversation length of 57 min (range: 33–103 min). The characteristics of the participating GPs are summarised in Table 2. Of the interviewed GPs, 58% were female. The average age was 52 years. Participants had worked for two to 43 years as a GP. Five participants held an additional qualification in palliative medicine. At the time of the interview, two participants were no longer practicing as GPs.

**Table 2:** Characteristics of GPs surveyed

Characteristics	Statistics			
**Age in years**	mean	52	range	30–79
**Gender**				
Female	n =	11	%	57.9
Male	n =	8	%	42.9
**Work experience as GP in years**	median	19	range	2–43
**No longer working as GP at time of interview**	n =	2	%	10.5
**Additional qualification in palliative medicine**				
Yes	n =	5	%	26.3
No	n =	14	%	73.7
**Practice type**				
Single-handed practice	n =	9	%	47.4
Group practice	n =	10	%	52.6
**Population size of practice location**				
Rural community (< 5,000 pop.)	n =	4	%	21.1
Small town (5,000–20,000 pop.)	n =	6	%	31.6
Large town (20,000–100,000 pop.)	n =	2	%	10.5
Urban centres (> 100,000 pop.)	n =	7	%	36.8
**German federal state**				
Bavaria	n =	3	%	15.8
Berlin	n =	2	%	10.5
Lower Saxony	n =	2	%	10.5
North-Rhine Westphalia	n =	2	%	10.5
Saxony	n =	1	%	5.3
Saxony-Anhalt	n =	1	%	5.3
Thuringia	n =	8	%	42.1

### Experienced frequency of requests for PAS and provided support of PAS

In summary, participants reported having experienced occasional requests for PAS during their time as GP. All but one interviewees had experienced at least one request in the course of their career. The most frequently mentioned number range from one to three requests (minimum: none in two years of professional experience; maximum: 15 in 43 years of professional experience).

One interviewee stated that she had intentionally provided PAS several times. Five others reported indirect involvement in PAS, for example, through the prescription of relevant medications without the intention of enabling a suicide, or through purely passive support of a person wishing to die who had contacted a right-to-die organisation.

The majority of participants indicated that they had not observed any changes in their practice following the Federal Constitutional Court's ruling from February 2020. Several participants assumed the onset of the COVID-19 pandemic in early 2020 to be the reason why the issue was not on the minds of many people, including some of the interviewed GPs. A few interviewees have noted an increase in requests or the need for information about AS and PAS from patients since the ruling. For a few patients, the ruling represented a groundbreaking, long awaited change.

### Contents of requests for PAS

The specific requests for PAS most often involved asking for a lethal drug, information on methods of suicide with medication, or general, unspecified requests for PAS. Other requests included referrals abroad for receiving AS or PAS, issuing a document for a right-to-die organisation, or prescribing swallowing therapy to enable the patient ingest lethal medication. In four cases, patients had already contacted a right-to-die organisation in advance.

*“She is planning to commit suicide by taking pills (…)*,* but she’s a bit worried that they might not work*,* (…) and she’s especially worried because she has difficulty swallowing*,* that she might not be able to get them down and end up in a persistent vegetative state. That was her big concern. […]”* (Interview U, passage 07)

### Sociodemographic backgrounds of patients requesting PAS

In some cases, participants provided detailed descriptions of patients who had requested PAS. According to these, many patients came from well-paid professions, had an academic education, a high degree of reflection, a strong sense of engagement and self-determination in their previous lives, and came from good financial and traditional family backgrounds. Patients who deviated from these attributes were also mentioned. However, the majority of patients requesting PAS were not described in detail.

*“She’s an old businesswoman*,* you know? Definitely not foolish. Very reflective*,* not poor either. […]”* (Interview U, passage 21)

*“[…] So*,* he was a very fine elderly gentleman*,* […] very polite*,* very friendly*,* […] very calm. Not someone who would wear his emotions on his sleeve.”* (Interview S, passage 17)

### Experienced motives for requests for PAS

Requests for PAS came predominantly from seriously ill patients, often in palliative situations. Most patients suffered from at least one form of cancer. In these cases, the GPs most frequently perceived physical weakness and associated immobility, increasing loss of autonomy, a sense of hopelessness, fear of further suffering, as well as severe pain and difficulty swallowing as main motives for the request for PAS.

Further diagnoses or diagnostic groups and the corresponding motives for requests for PAS as perceived by the interviewees can be found in Table 3. Not in all cases was the request for PAS motivated by a medical diagnosis. A frequently mentioned group of patients is characterised primarily by their advanced age. In these patients, the prospect or the experience of loneliness, a feeling of senselessness, weariness with life as well as the need for care, and immobility often drove the wish to die. Another patient without a severe medical diagnosis asked for PAS driven by homelessness, financial poverty, loneliness, and the feeling of senselessness.

Further cases reported by the interviewees dealt with patients suffering from depression, severe COPD, degenerative motor neuron diseases, dementia, and chronic pain syndrome. In addition to somatic or psychological complaints, motives restricting personal independence like the loss of autonomy, loss of control, or immobility as well as loneliness and senselessness were mentioned repeatedly as motives driving the wish for PAS.

*“It’s often the loss. The loss […] of independence. It’s pain. It’s […] the hopelessness and […] especially the loss […] of autonomy. Like: ‘I rely on someone always being there. I can no longer go to the bathroom alone.’ That’s what it is*,* […] I would guess that the loss of autonomy is certainly 60%.”* (Interview L, passage 33)

**Table 3:** Diagnoses and complaints related to the request for PAS as perceived by the interviewed GPs with exemplary quotations

Type of diagnosis and main complaints (number of interviews in which mentioned)	Quote from the qualitative data
**Cancer (> 10)**	
Physical weakness (partly cachexia) Immobility Increasing care requirements Loss of autonomy Loneliness Hopelessness Mental stress Fear of further suffering (e.g. pain, loss of control) Pain Shortness of breath Swallowing difficulties	*“[…] And I think the wish […] of many patients when they express a desire for assisted suicide is also that they simply don’t want to suffer pain*,* don’t want to suffer shortness of breath*,* […] can’t bear loneliness. So*,* it’s really about overcoming this sense of hopelessness as well.”* (Interview R, passage 03)
**Advanced age (min. 9)**	
Expected or experienced loneliness Senselessness Weariness with life Need for care Immobility	*“And an old gentleman*,* same story– wife died*,* has nothing left*,* children far away*,* doesn’t want to live anymore– […] although in his case […] well*,* the problem is: he’s perfectly healthy.”* (Interview C, passage 20) *“[…] This is an 85-year-old gentleman. He’s been a patient since I took over the practice*,* so I’ve known him for 20 years. His second wife has now died. She was much younger than him. And he says he actually didn’t want to go through this again after his first wife died. And now she’s dead too*,* and he said*– *sounds silly*– *that he had deliberately married someone younger so he wouldn’t have to experience it again. Now it’s happened anyway. […] For him*,* it is definitely the expected loneliness. It doesn’t exist yet*,* but it’s going to happen. He also has two sons and a daughter. And they all live nearby. But of course*,* they live their own lives*,* and it’s an expected loneliness. […]”* (Interview K, passages 19–23)
**Depression (min. 5)**	
Mental stress Psychosomatic pain Insomnia	*“[…] Surprisingly*,* in my career*,* it’s been more patients with mental health issues. Rarely patients who belong to the oncological-palliative spectrum*,* but really people who just say everything is too exhausting*,* and they would like to go to Switzerland and ask if I could help them with that. […]”* (Interview B, passage 03)
**Severe/burnt-out COPD (min. 4)**	
Shortness of breath Immobility Anxiety Pain	*“Yes*,* well*,* he had severe COPD*,* and at first*,* he didn’t fit into the patient group I would have expected this from. […] I hadn’t initially perceived him that way. And the fact that someone with severe COPD would have a death wish due to this condition*,* I wouldn’t have thought of that before*,* to be honest. In his case*,* he was about 70 (…) and was practically out of treatment options*,* as they say. Already under maximum therapy*,* he had experienced several severe exacerbations each year*,* with repeated stays in intensive care and a difficult recovery afterward. He was simply short of breath*,* even when just making movements while sitting in a chair*,* and he no longer found life worth living.”* (Interview P, passage 23)
**Degenerative motor neuron disease (min. 2)**	
Loss of autonomy Immobility	*“[…] her radius is simply getting smaller. So*,* she can only walk with a walking aid and […] well*,* I think with her*,* […] the loss of autonomy is very strong. […]”* (Interview S, passage 23)
**Dementia (min. 1)**	
Loss of control	*“[…] it was a professor who had Alzheimer’s and noticed it. And before he had completely declined*,* he expressed this wish. […]”* (Interview R, passage 15)
**Chronic pain syndrome (min. 1)**	
Pain Immobility Hopelessness	*“[…] Well*,* her legs are always so overheated and aching and hot and stinging and burning and she always has a fan on her feet all day long*,* and packs herself full of cool packs*,* and then has got […] cold blisters*,* which then hurt even more. And she simply has no more courage to face life because […] there is simply no adequate therapy […]”* (Interview G, passage 04)
**No medical diagnosis (min. 1)**	
Loneliness Financial poverty Homelessness Senselessness	*“[…] a completely unsuccessful life […] without stable relationships*,* constantly searching*,* trying to buy people […] and thus totally impoverished herself […]. And when there was nothing left to get*,* there was no one left. […] she wanted to end that kind of life […].”* (Interview N, passage 19)

### Implicit requests for PAS: frequency, barriers, and handling

Several participants reported they had once or multiple times felt the topic of PAS to be present but not spoken of during a consultation with a patient. The suspected barriers for patients included fear of discussing it, not wanting to burden their doctor with such a sensitive issue, religious aspects, or ignorance about the possibility of PAS. Most interviewees chose not to address the topic on their own in such situations. Various reasons for this decision were provided:

- time constraints;
- the concern of putting the idea into the patient’s mind by bringing up this possibility;
- the feeling of not being able to help if there was a genuine request for PAS;
- the assessment that the patient’s illness was too advanced to consider PAS, since natural death was immanent;
- the belief that the initiative for this existential topic must come from the patients themselves;
- reluctance to engage in a difficult conversation.

One GP reported always addressing the topic when she sensed its presence. In her experience, these conversational interventions often lead to relief and a withdrawal from the desire for PAS.

*" […] And at that moment*,* it also […] apparently goes away a little […] yes? When you have shared it with someone*,* yes? […] So*,* that’s really wonderful*,* it brings relief. And then maybe it’s not so pressing anymore. […] I’m just realising this now as we talk about it.”* (Interview N, passage 51)

### Experienced frequency and motives of vague wishes for assisted dying

In contrast to the occasional requests for PAS, there are vague wishes for assistance in dying. In these cases, PAS is not specifically requested, but a verbal expression of the wish for help in dying is made. Most participants indicated that such expressions are frequently made to them. It is worth noting that participants consistently differentiated between non-specific expressions of wishes for assistance in dying and explicit requests for PAS on their own initiative. Based on their descriptions, patients with vague wishes for help in dying can be grouped as follows: patients of advanced age, depressed patients, overburdened patients, patients with a high symptom load (often multimorbide or palliative), and dying patients.

*“Well*,* sometimes you do get the usual requests*,* like if you can do something to make it go faster or something. However*,* it’s […] never a consistent statement*,* and you can catch those people quickly if you provide proper palliative care. […] These requests are definitely not lasting. […] if there are interventions from family or the church or wherever*,* […] it quiets down again. […]”* (Interview U, passages 07–09)

## Discussion

In the HAPASS study we interviewed 19 German GPs on their perspectives on PAS. Nearly all participants reported having received explicit requests for PAS at some point in their clinical practice, with the highest frequency occurring among elderly and oncology patients. In most cases, the inquiries requested a lethal agent or information about the fatal dosage of the patient’s own medication, or they involved unspecified requests for PAS. From individual descriptions of inquiring patients emerges a profile of predominantly well-educated, financially secure individuals from traditional family backgrounds. Participants predominantly identified the loss of autonomy and functional independence as primary motivators for PAS consideration. Only one GP reported multiple instances of direct suicide assistance. Five others described indirect or unintentional participation. Most interviewees did not observe an increase in requests following the 2020 ruling by the Federal Constitutional Court [1].

### Frequency of requests for PAS in general practice

The interviews revealed that requests for PAS in general practice in Germany happen occasionally. It is likely that GPs will be confronted with such a request once or several times over the course of their career, as both German [8] as well as international studies have suggested [12, 14, 26]. The majority of participants did not report an increase in demand for AS following the Federal Constitutional Court’s ruling in February 2020. It should be considered that at the time of ruling, it was primarily noticed and discussed by professional circles, rather than by the public, as media coverage and discussion were dominated by the emerging COVID-19 pandemic. International reviews show that in other countries, the longer AS and PAS were legalised, the more requests for it increased [27, 28].

### Involvement of GPs in PAS

Very few of the interviewed GPs had intentionally participated in PAS. On one hand, this supports the findings of a recent study which found through the analysis of death certificates of cases of AS in Munich that GPs rarely play a role in PAS that are facilitated by right-to-die organisations [11]. On the other hand, our interviews revealed that some of the interviewed GPs were a contact point on the patient’s path to PAS, for example, with a right-to-die organisation. This expands the scope of the Munich study [11] which was unable to determine from death certificates whether GPs or other physicians or therapists were involved in the process leading up to PAS. Additionally, both in the Munich study as well as in international research there are reports of unintentional involvement of GPs or other physicians in PAS, for example through the prescription of symptom-relieving medications [8, 15, 27]. These findings suggest that there might be a considerable high dark number of PAS with the participation of GPs.

In a European comparison, based on the existing literature and the findings from the interviews the role of German GPs in PAS appears to be most comparable to the role of Swiss GPs. They receive requests for PAS, but they rarely respond to them in the form of actual suicide assistance. German GPs may not refer as often to right-to-die organisations as the Swiss do [16, 17]. This is presumably because both right-to-die organisations as well as the topic of PAS itself are not yet very well established in Germany. The biggest difference can be found in the comparison to Dutch GPs, who act as the most frequent performers of PAS as well as euthanasia in the Netherlands [12–14].

### Barriers for GPs to address the topic of PAS

Most of the interviewed GPs did not proactively address the issue of PAS with patients, even when they sensed it was unspoken but present in the room. The reasons given included primarily a lack of time, feeling overwhelmed, and concerns about the negative impact of such conversations. The first two reasons suggest structural barriers and a need for further training or reflection in order to handle death wishes and requests for PAS responsibly and professionally. Concerns about negative impacts of such conversations could be driven by the fear of giving patients the impression that their doctor sees an indication for PAS in their case. However, there is a known preventative effect of addressing potential death wishes, including suicidality [29]. One interviewee reported a relieving experience after addressing an unspoken wish for PAS. In the S1 guideline recommendations of the DEGAM (German Society for General Practice and Family Medicine) for dealing with requests for PAS in general practice [30], GPs are encouraged to address such wishes, even if they are implicit. In light of the concerns raised, appropriate wording is essential.

### Perceived motives for requests for PAS

Based on the existing literature, we expected that a large proportion of patients requesting PAS would suffer from haematological-oncological conditions [14, 27, 28, 31–33]. Our findings confirmed this assumption. However, the interviews also highlighted that not all patients requesting PAS had an underlying illness as the motivation for their request. Very elderly patients who were not particularly ill made a substantial portion of the requests. This result replicates findings from other studies [31, 33, 34], even though old age alone does not necessarily meet the criteria of a terminal illness or unbearable suffering that are mandatory to be eligible for PAS in other countries. Furthermore, both the lead author and one interviewee were surprised by repeated reports of COPD patients. One explanation for why this patient group might request PAS more frequently than some other chronically ill patient groups may lie in the motivations, as identified by the GPs, that drove patients to request PAS (Table 3). In addition to physical or mental symptoms that could be targets for therapeutic intervention, there were also motivations touching on the overarching aspects of independence and autonomy in nearly all groups of diagnoses. These include, for example, (anticipated) care needs and restrictions in physical mobility due to weakness and immobility. Especially the latter is often the case for oxygen-dependent patients with end-stage COPD. The loss of autonomy and dignity, as well as a declining quality of life are also identified as main reasons underlying the decision for PAS or euthanasia in scientific reviews on assisted dying [27, 28].

The occasional detailed descriptions of patients requesting PAS present a picture of predominantly socially well-off, independent, engaged, and reflective individuals. Similar descriptions are found in international studies [15, 27, 28, 32, 35]. It is conceivable that this group in particular experiences the (anticipated) loss of autonomy and independence as well as loneliness and hopelessness as especially detrimental to their quality of life, and may consider PAS the last option for a self-determined action.

### Practical implications and outlook

Awareness of prominent characteristics and common motives can be helpful in practice for identifying potentially vulnerable patients and better understanding the requests for PAS that are presented.

The motives for considering PAS are multifaceted. Not only terminally ill persons request PAS. Besides palliative medical situations, advanced age and associated aspects such as loneliness, care needs, and being tired of life are driving people to consider the path of PAS. In light of demographic changes and the growing shortage of skilled workers in the care sector, the results reported here suggest that requests for AS and PAS may become more frequent in the future. Therefore, and because of the existential nature of the topic, it seems reasonable for GPs to engage with it, even if it is not a common reason for consultation. The likelihood of being confronted with it in the future seems high.

### Vague wishes for assistance in dying

All interviewees reported that they receive vague wishes for assistance in dying significantly more frequently than explicit requests for PAS. Patients expressing such wishes were predominantly described as severely symptom-burdened or distressed. These descriptions, along with the exemplary interview excerpt, suggest that GPs interpret these wishes as demands for improved treatment of current complaints rather than an actual directive for assistance in dying. Such an understanding is also reflected in the international consensus definition of the wish to hasten death, which defines this wish as a reaction to suffering [36]. This incidental finding from the interviews suggests that GPs perceive and interpret expressed wishes for assistance in dying communicated to them in a nuanced manner. Further research could address the factors that influence this differentiation.

### Strengths and limitations

To our knowledge, this study is the first one since the ruling of the Federal Constitutional Court in 2020 to focus in depth on German GPs’ perspectives on PAS. As a qualitative study with an iterative process, the HAPASS study is open to a wide range of aspects arising that a questionnaire with predefined questions could not detect. Thus, a strength of this study lies in its explorative character, which could not have been achieved with a quantitative study design. The results of this study can serve as a basis for planning a quantitative study to generate representative and generalisable results.

The limitations of the study arise from the small amount of experiences with PAS. Since these limited experiences date back different lengths of time, different regulations concerning the law and the Medical Code of Conduct apply to them. Theoretical saturation was achieved in several aspects, but not in all. This is most likely due to the heterogenity of individual experiences and attitudes that characterise this topic.

The results of this study concerning vague wishes for help in dying and the sociodemographic characterisation of patients should be considered superficial by-products, as these aspects were not the focus of the investigation and were therefore not further explored in the interviews.

Furthermore, there remains a possibility of bias due to the chosen recruitment strategies for interview partners. For feasibility reasons, we recruited a portion of the participants as a convenience sample from the connections of the Institute of General Practice and Family Medicine in Jena. Other interviewees volunteered after a call for participation in a journal. Due to volunteer bias, there is a possibility that particularly strong opinions among respondents may be overrepresented, while the average position may be underrepresented. However, the data do not suggest this. Additionally, selection bias and sampling bias must be considered, meaning the data are not generalisable. However, this was not the objective of this qualitative work. For the goal of openly exploring a previously unresearched topic, these biases are therefore weighted as less severe.

## Conclusion

PAS is an occasional reason for consultation in German general practices. Although there does not appear to have been a significant increase in requests for PAS since the German Federal Constitutional Court’s ruling in 2020, it is likely that with demographic change, increasing shortage of nurses, and social acceptance the impact of this issue will increase in the future. Until now, German GPs appear very rarely to assist with suicide intentionally; however, unintentional assistances happen and can be identified only through reflection afterwards as actual PAS. Overall, the GPs’ role seems to be more of an advisory, informative, caring rather than an actively assisting one. This role also corresponds to the content of patients’ requests, who mainly ask for lethal medication or information about lethal doses of their own medication. Patients who present with requests for PAS are often either seriously ill or of advanced age. They tend to be described by their GPs as reflective, well-educated and self-determined persons. From the GPs’ point of view, a frequently occurring motive for the desire for PAS regardless of the diagnosis is the perceived (impending) loss of self-determination and autonomy. Especially when a loss of this kind is immanent or incipient, situations may arise where patients do not bring up the topic of PAS directly, but imply it in their remarks. The study shows that GPs tend not to address implicit requests for PAS on their own initiative. The main reasons for this are both structural barriers as well as fear and uncertainty about how to deal with this issue.

## Electronic supplementary material

Below is the link to the electronic supplementary material.

Supplementary Material 1

## Data Availability

The pseudonymised transcripts used and/or analysed during the current study are available from the corresponding author on reasonable request.

## Abbreviations

AS: Assisted suicide
COPD: Chronic obstructive pulmonary disease
GP: General practitioner
HAPASS: Hausärztliche Perspektiven auf den assistierten Suizid (GPs’ perspectives on (physician-)assisted suicide)
PAS: Physician-assisted suicide
