# Supplementary material for: Requests for physician-assisted suicide in German general practice: frequency, content, and motives– a qualitative analysis of GPs’ experiences
Source: BMC Prim Care. 2025 Apr 23;26:122. doi: 10.1186/s12875-025-02830-0 (PMC12016426; doi:10.1186/s12875-025-02830-0)
Supplement: Supplementary file 1 — Supplementary Material 1 [file 12875_2025_2830_MOESM1_ESM.docx]

**Interviewleitfaden**

1. **Erfahrungen**

Bitte schildern Sie Ihre bisherige Auseinandersetzung mit dem Urteil des Bundesverfassungsgerichts vom 26.02.2020, assistiertem Suizid und Sterbehilfe.

Welche persönlichen oder beruflichen Vorerfahrungen haben Sie mit diesen Themen, insbesondere mit dem assistierten Suizid?

Hat sich in Ihrer Praxis seit dem Urteil des Bundesverfassungsgerichts etwas verändert? Glauben Sie, dass sich zukunftig etwas ändern wird?

 Wie erleben Sie in Ihrer Praxis die Nachfrage nach Suizidassistenz:

- Wie häufig ist eine solche Nachfrage schon an Sie als Hausarzt/Hausärztin herangetragen worden?
- Welche Gründe/Motive haben Sie seitens der Patient:innen für Nachfragen nach Suizidassistenz wahrgenommen?
- Hat sich seit dem Urteil des Bundesverfassungsgerichts etwas hinsichtlich der Nachfragen verändert (z.B. Häufigkeit)?

1. **Einstellungen**

Wie sind Sie dem Thema Suizidassistenz gegenüber eingestellt?

Welche positiven Aspekte sehen Sie darin, dass Suizidassistenz möglich ist?

Welche Bedenken haben Sie in Bezug auf das Thema Suizidassistenz?

Unter welchen Umständen könnten Sie sich vorstellen, an einem assistierten Suizid mitzuwirken, und wann nicht?

In welcher Funktion könnten Sie sich vorstellen, an einem assistierten Suizid mitzuwirken (z.B. Beratung, Begutachtung – sofern notwendig, organisatorische Vorbereitungen, Verschreibung eines tödlichen Medikaments, Begleitung bei der Durchführung)?

 Wie sehen Sie die hausärztliche Rolle in dieser Thematik?

1. **Erfordernisse (für eine verantwortungsvolle Umsetzbarkeit des Urteils)**

Wer (im Sinne von: welche Professionen/Personen) sollte Ihrer Meinung nach im Prozess rund um eine Suizidassistenz beteiligt sein?

Wo sehen Sie die hausärztliche Rolle in diesem Prozess? Welche Aufgaben kann oder sollte die Hausärztin/der Hausarzt übernehmen, welche lieber nicht?

Welche Hürden oder Schwierigkeiten können Sie sich bei der Umsetzung einer Suizidassistenz (vom ersten Gespräch bis zur Durchführung) insbesondere im hausärztlichen Kontext vorstellen bzw. haben Sie schon selbst erlebt?

Wie könnte diesen Hürden/Schwierigkeiten konstruktiv begegnet/entgegengewirkt werden?

1. **Sonstiges**

Was glauben Sie, wie eine Fragebogenstudie zu dem Thema von Hausärzt:innen angenommen wird?

Haben Sie weitere Gedanken zu dem Thema, die Sie noch mitteilen möchten?

Was wäre aus Ihrer Sicht interessant/relevant in weiteren Studien zum Thema Suizidassistenz zu fragen/untersuchen?

**Interview Guide**

1. **Experiences**

Please describe your previous engagement with the verdict of the Federal Constitutional Court of 26 February 2020, assisted suicide and euthanasia.

What previous personal or professional experience do you have with these topics, in particular with assisted suicide?

Has anything changed in your practice since the verdict of the Federal Constitutional Court? Do you think anything will change in the future?

How do you experience the demand for assisted suicide in your practice?

- How often have you received requests vor assisted suicide in your role as a GP?
- What reasons/motives have you perceived on the part of patients asking for assisted suicide?
- Has anything changed in terms of requests since the verdict of the Federal Constitutional Court (e.g. frequency)?

1. **Attitudes**

What is your attitude towards assisted suicide?

What positive aspects do you see in the fact that assisted suicide is possible?

What concerns do you have about assisted suicide?

Under what circumstances could you imagine participating in an assisted suicide and when not?

In what capacity could you imagine participating in an assisted suicide (e.g. counselling, assessment - if necessary, organisational preparations, prescribing a lethal medication, accompanying the suicide)?

How do you see the role of GPs in this area?

1. **Requirements (for responsible implementation of the verdict)**

Who (meaning which professions/persons) should be involved in the process of assisted suicide in your opinion?

Where do you see the GP's role in this process? Which tasks can or should the GP take on, and which would you rather not?

What hurdles or difficulties can you imagine or have you already experienced in the implementation of suicide assistance (from the first discussion to implementation), particularly in the GP context?

How could these hurdles/difficulties be constructively countered/counteracted?

1. **Other**

How do you think a questionnaire study on this topic would be received by GPs?

Do you have any other thoughts on the topic that you would like to share?

What do you think would be interesting/relevant to ask/investigate in further studies on the topic of assisted suicide?
